# Supplementary material for: Active site metals mediate an oligomeric equilibrium in Plasmodium M17 aminopeptidases
Source: J Biol Chem. 2020 Dec 17;296:100173. doi: 10.1074/jbc.RA120.016313 (PMC7948507; doi:10.1074/jbc.RA120.016313)
Supplement: Supplementary Figures and Tables [file mmc1.pdf]

A Metal Dependent Dynamic Equilibrium Mediates Oligomerization of M17 Aminopeptidases from *Plasmodium falciparum* and *Plasmodium vivax*

Tess R. Malcolm[1], Matthew J. Belousoff[1], Hariprasad Venugopal[2], Natalie A. Borg[3,4], Nyssa Drinkwater[1], Sarah C. Atkinson[3,4] & Sheena McGowan[1*]

[1]Infection & Immunity Program, Monash Biomedicine Discovery Institute and Department of Microbiology, Monash University, Clayton, Victoria, 3800, Australia
[2]Ramacciotti Centre for Cryo-Electron Microscopy, Monash University, Clayton, Victoria, 3800, Australia
[3]Infection & Immunity Program, Monash Biomedicine Discovery Institute and Department of Biochemistry and Molecular Biology, Monash University, Clayton, Victoria, 3800, Australia
[4]Immunity and Immune Evasion Laboratory, Chronic Infectious and Inflammatory Diseases Research, School of Health and Biomedical Sciences, RMIT University, Bundoora, Victoria, 3083, Australia

SUPPORTING INFORMATION

Supplementary Table S1. Hydrodynamic properties of *Pv*-M17 and *Pf*A-M17

Supplementary Table S2. *Pv*-M17Δ125-151 X-ray crystal structure data collection and refinement statistics.

Supplementary Table S3. *Pv*-M17 cryo-EM data collection and model statistics

Supplementary Table S4. *Pv*-M17 primers used to generate metal-binding mutant and N-terminal loop mutant

Supplementary Figure S1. Analytical ultracentrifugation sedimentation equilibrium of *Pf*A-M17 (W525A + Y533A)

Supplementary Figure S2. Analytical ultracentrifugation sedimentation equilibrium of *Pv*-M17 in the presence of 50 μM Mn^{2+}.

Supplementary Figure S3. Sequential alignment with mapped secondary structure of *Pf*A-M17 (PDB ID: 3KQZ) and *Pv*-M17.

Supplementary Figure S4. Active site metal ion coordination by *Pv*-M17 and *Pf*A-M17

Supplementary Figure S5. Resolution of *Pv*-M17 tetramer and hexamer cryo-EM models

Supplementary Figure S6. Size exclusion chromatography trace of *Pv*-M17 forming a tetrameric species in presence of 100 mM EDTA

**Supplementary Table S1. Hydrodynamic properties of *Pv*-M17 and *Pf*A-M17**						
	**M**[a]	$s_{20,w}$[b]	**M_1**[c]	**M_2**[d]	**f/f_0**[e]	**Oligomeric state**
***Pv*-M17 WT + Mn**	60.4	12.5	316	365.4	1.34	hexamer
***Pf*A-M17 WT + Mn**	58.6	12.2	332	-	1.01	hexamer
***Pf*A-M17 (W525A + Y533A)**	58.6	3.3	56.0	59.4	1.45	monomer

[a] Relative molecular weight calculated from the amino acid sequence
[b] Standardized sedimentation coefficient taken from the ordinate maximum of the *c*(*s*) distribution best fits
[c] Molar mass determined from the ordinate maximum of *c*(*M*) distribution best fits (data not shown)
[d] Molar mass determined from the sedimentation equilibrium analysis
[e] Frictional coefficient calculated from $s_{20,w}$ using the $\bar{v}$ method employing SEDNTERP

**Supplementary Table S2.** ***Pv*****-M17Δ125-151 X-ray crystal structure data collection and refinement statistics (PDB ID: 6WVV).**	
**Data Collecting and Processing**	
Diffraction Source	MX2 beamline, AS
Wavelength (Å)	0.953660
Temperature (K)	100
Detector	Eiger
Crystal-to-detector distance (mm)	360.613
Space group	P1 21 1
a, b, c (Å)	116, 201, 165
α, β , γ (˚)	90, 90, 106
Mosaicity (˚)	0.10
Resolution range (Å)	48.95 – 2.29 (2.33 – 2.99)
Total no. of reflections	1461300 (62273)
No. of unique reflections	327356 (15478)
Completeness (%)	99.7 (95.3)
Multiplicity	4.5 (4.0)
$\langle I/\sigma(I) \rangle$	0.54 (0.47)
R_{meas} (%)	0.174 (1.431)
Rpim	0.107 (0.897)
**Refinement Statistics**	
R-work	0.2054
R-free	0.2435
RMSD Bond Lengths (Å)	0.002
RMSD Bond Angles (˚)	0.4
Ramanchandran favored (%)	96
Ramachandran outliers (%)	0.3
Clashscore	2.0
Values in parenthesis are for outer shell	

Supplementary Table S3. *Pv*-M17 cryo-EM data collection and model statistics		
**Data collection**	**Hexamer (PDB ID: 7K5K)**	**Tetramer**
Particles	32,312	16,127
Pixel size	1.06	1.06
Voltage (kV)	300	300
Electron dose (e/Å^2)	63	105
**Refinement**		
CC_{map_model}	0.84	
**Model Quality**		
RMSD Bond length (Å)	0.005	
RMSD Bond angles (˚)	0.633	
Ramachandran favored (%)	91.41	
Ramachandran outliers (%)	0.09	
Ramachandran allowed (%)	8.50	
Rotamer outliers (%)	5.01	
C-beta deviations (%)	0	
Clashscore	7.26	

**Supplementary Table S4.** ***Pv*****-M17 primers used to generate metal-binding mutant and N-terminal loop mutant**	
**Mutation**	**Primers**
D395A	Forward 5' – GGT ATT ACG TTT GCG TCC GGT GGC - 3'<br>Reverse 5' – GCC ACC CGA CGC AAA CGT AAT ACC - 3'
E477L	Forward 5' – AAC ACC GAT GCC GCA GGC CGT TT - 3'<br>Reverse 5' – AAA CGG CCT GCG GCA TCG GTG TT - 3'
*Pv*M17Δ125-151	Forward 5' – GCG CAG ATC AAG ATT AAT TCT TCG – 3'<br>Reverse 5' – GCT GTT CAG CAA GAA AAC GAT- 3'

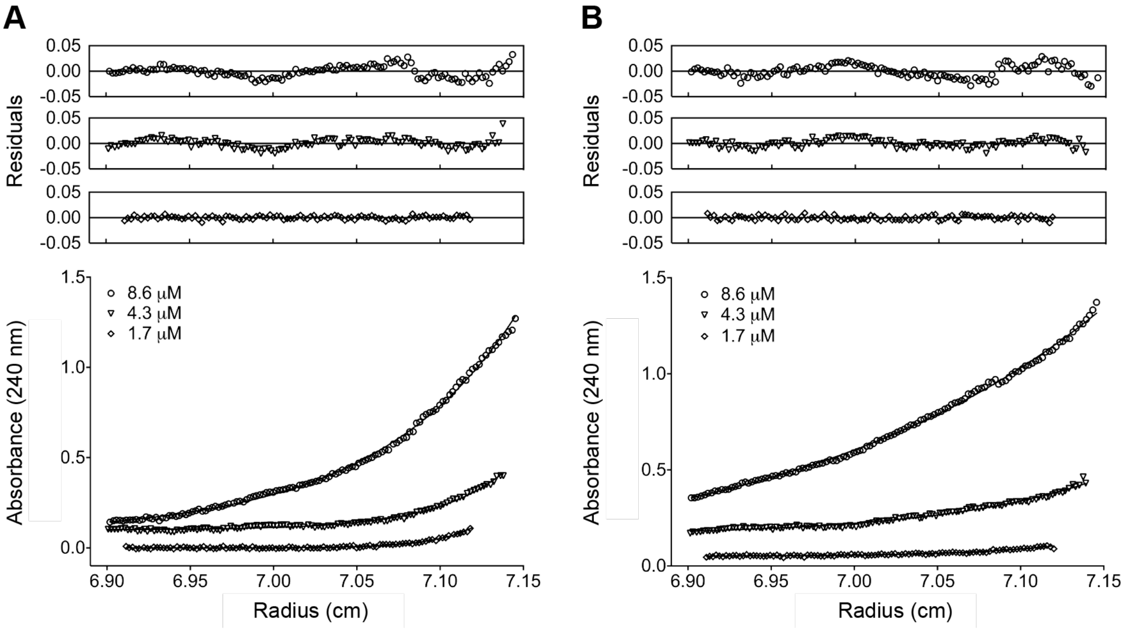

Supplementary Figure S1. Analytical ultracentrifugation sedimentation equilibrium of *Pf*A-M17 (W525A + Y533A). Global fit analysis of PfA-M17 (W525A + Y533A) at 8.6 µM (circle), 4.3 µM (triangle) and 1.7 µM (diamond) and corresponding residuals at two different speeds (A)12,000 rpm and (B) 18,000 rpm. Single species model yielded a molecular weight of 59.4 kDa indicating that PfA-M17 (W525A + Y533A) is a monomer in solution.

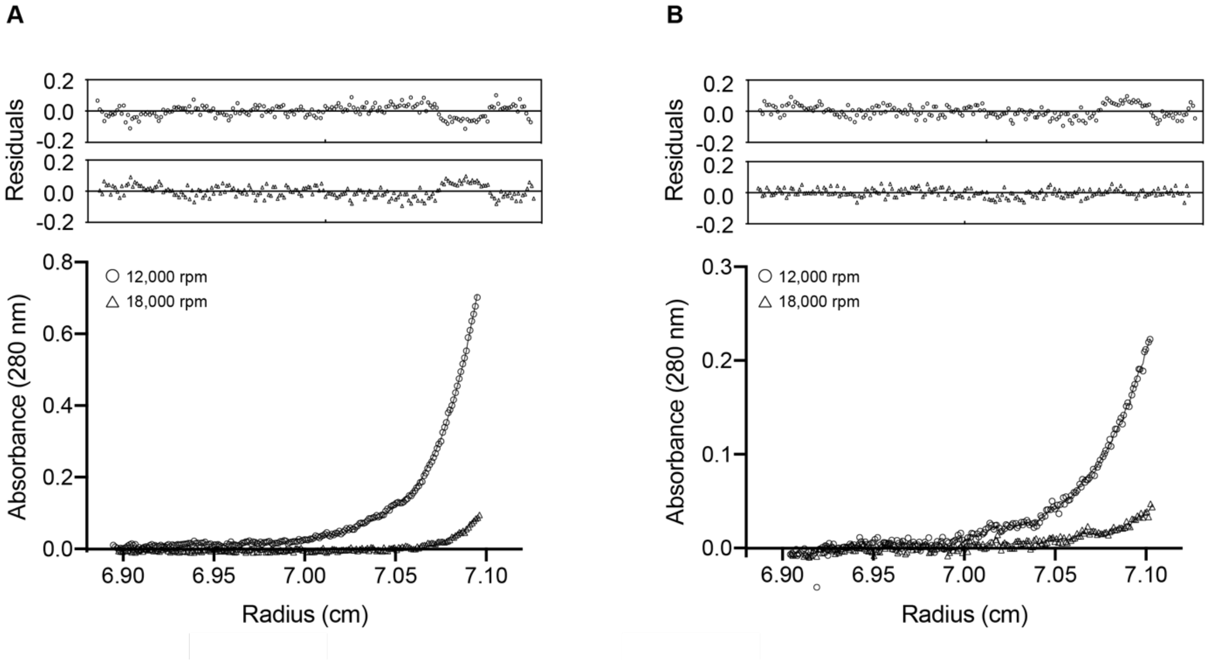

Supplementary Figure S2. Analytical ultracentrifugation sedimentation equilibrium of *Pv*-M17 in the presence of 50 μM Mn^{2+}. Global fit analysis of Pv-M17 at 8.3 μM (A) and 4.2 μM (B) and corresponding residuals at two different speeds 12,000 rpm (blue circles) and 18,000 rpm (green triangles). Single species analysis yielded a molecular weight of 365.4 kDa and indicated that *Pv*-M17 is a hexamer in solution supplemented with 50 μM Mn^{2+}.

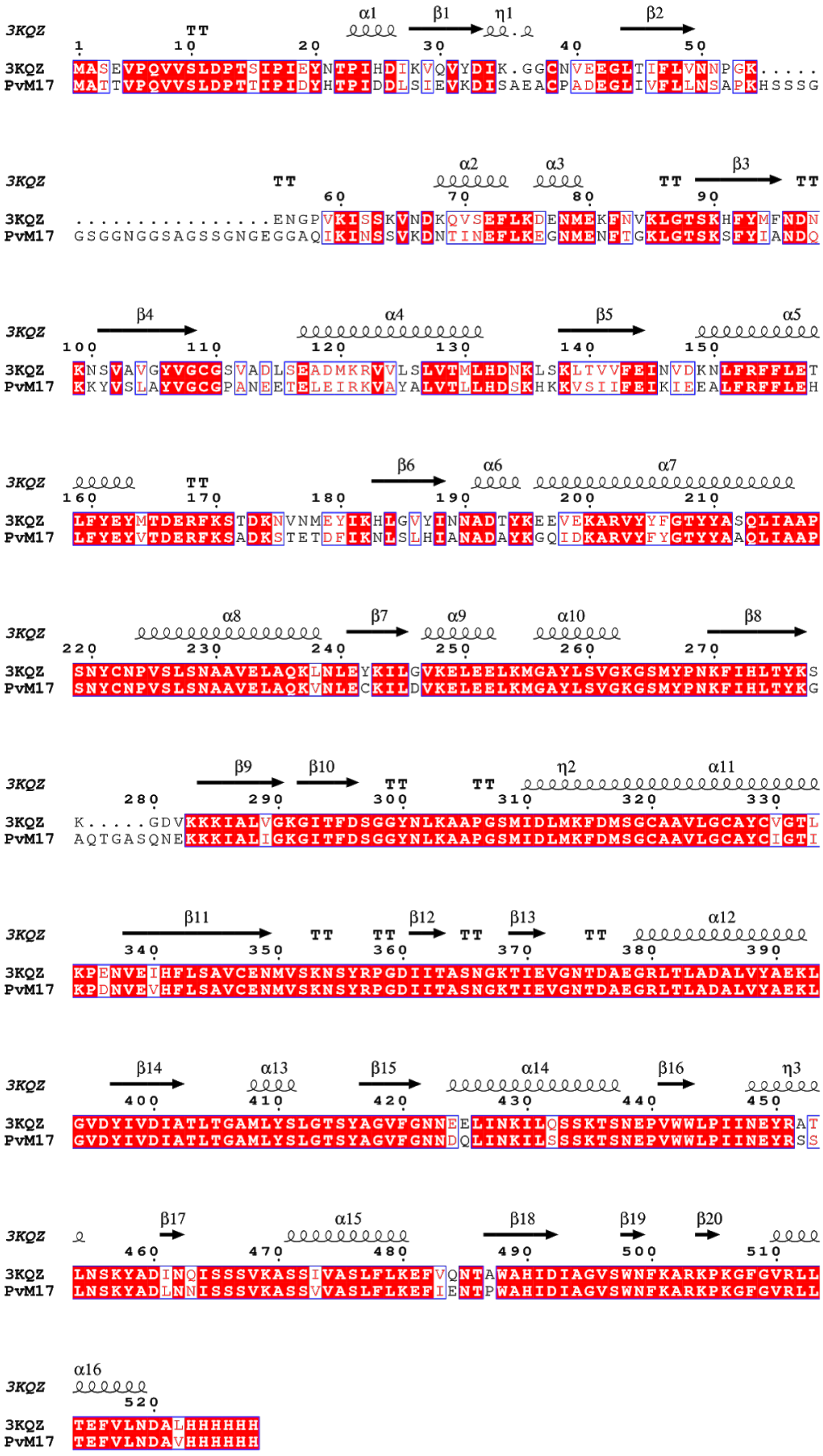

Supplementary Figure S3. Sequential alignment with mapped secondary structure of *Pf*A-M17 (PDB ID: 3KQZ) and *Pv*-M17. Sequence alignment carried out in ESPript 3. Sequence is highly conserved between *Pf*A-M17 and *Pv*-M17, with the exception of a large N-terminal insertion in the *Pv*-M17 sequence.

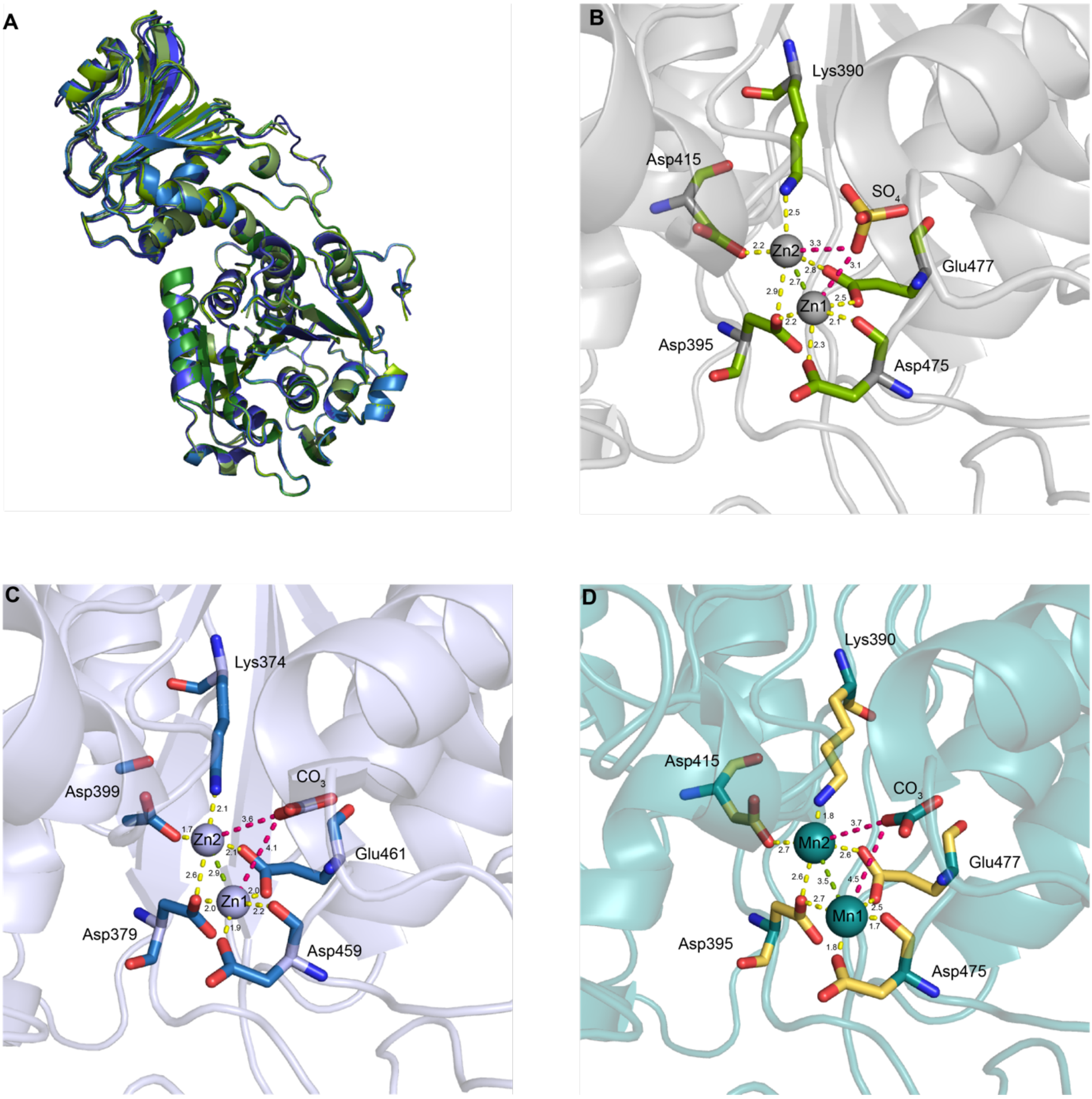

Supplementary Figure S4. Active site metal ion coordination by *Pv*-M17 and *Pf*A-M17. **(A)** Overlay of individual chains that compose the *Pv*-M17 hexamer crystal structure (PDB ID: 6WVV). The C-terminal structure is highly conserved between chains. The N-terminal is more dynamic and varies slightly between chains. **(B)** *Pv*-M17 crystal structure active site with two Zn ions and one sulfate ion bound. Zn1 is coordinated by Asp395, Asp475 and Glu477 and Zn2 is bound by Lys390, Asp395, Asp415 and Glu477. Distances (Å) between metal ions and protein residues are shown in yellow, between Zn1 and Zn2 shown in green, and between the metal ions and sulfate ion as pink. **(C)** *Pf*A-M17 crystal structure (PDB ID: 3KQZ) active site with two Zn ions and one carbonate ion bound. Zn1 is coordinated by Asp379, Asp459 and Glu461. Zn2 is coordinated by Lys371, Asp379, Asp399 and Glu461. Distances (Å) between metal ions and protein residues are shown in yellow, between Zn1 and Zn2 shown in green, and between the metal ions and carbonate ion as pink. **(D)** *Pv*-M17 cryo EM hexamer active site with two Mn ions and one carbonate ion bound. Mn1 is coordinated by Asp395, Asp475 and Glu477 and Mn2 is bound by Lys390, Asp395, Asp415 and Glu477. Distances (Å) between metal ions and protein residues are shown in yellow, between Mn1 and Mn2 shown in green, and between the metal ions and carbonate ion as pink.

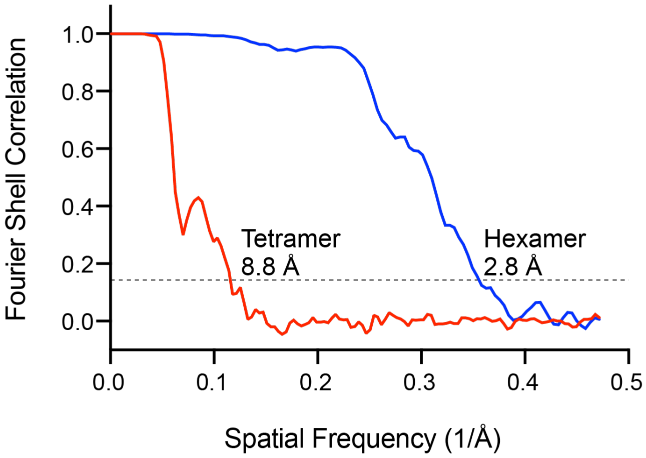

Supplementary Figure S5. Resolution of *Pv*-M17 tetramer and hexamer cryo-EM models. Fourier shell correlation (FSC) vs spatial resolution for the maps corresponding to *Pv*-M17 tetrameric species (red line; 8.8 Å) and *Pv*-M17 hexameric species (blue line; 2.8 Å). Dashed line shows the gold-standard FSC threshold at 0.143.

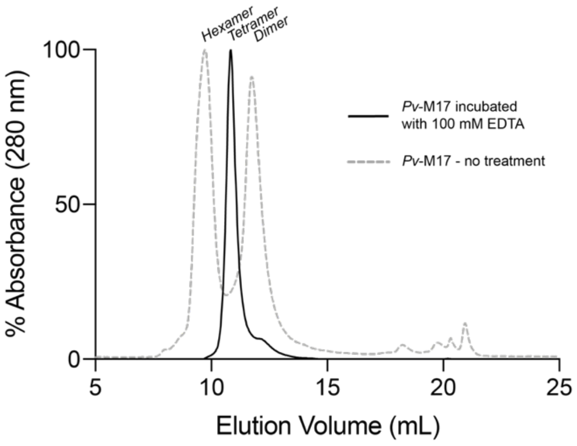

Supplementary Figure S6. Size exclusion chromatography trace of *Pv*-M17 forming a tetrameric species in presence of 100 mM EDTA. *Pv*-M17 forms a single tetrameric species (black line) when incubated with 100 mM EDTA. EDTA-spiked tetramer sample was used to generate the cryo-EM tetramer map. *Pv*-M17 with no treatment (grey dashed line) elutes as a mix of species, and is predominantly hexameric and dimeric.
